# Deep sympatric mtDNA divergence in the autumnal moth (*Epirrita autumnata*)

**DOI:** 10.1002/ece3.434

**Published:** 2013-01-10

**Authors:** Kjersti S Kvie, Silje Hogner, Leif Aarvik, Jan T Lifjeld, Arild Johnsen

**Affiliations:** 1 Natural History Museum, University of OsloP.O. Box 1172, Blindern, N-0318, Oslo, Norway; 2 Department of Basic Sciences and Aquatic Medicine, Norwegian School of Veterinary ScienceP.O. Box 8146 Dep, N-0033, Oslo, Norway

**Keywords:** DNA barcoding, *Epirrita autumnata*, mtDNA, nDNA, selective sweep, *Wolbachia*

## Abstract

Deep sympatric intraspecific divergence in mtDNA may reflect cryptic species or formerly distinct lineages in the process of remerging. Preliminary results from DNA barcoding of Scandinavian butterflies and moths showed high intraspecific sequence variation in the autumnal moth, *Epirrita autumnata*. In this study, specimens from different localities in Norway and some samples from Finland and Scotland, with two congeneric species as outgroups, were sequenced with mitochondrial and nuclear markers to resolve the discrepancy found between mtDNA divergence and present species-level taxonomy. We found five COI sub-clades within the *E. autumnata* complex, most of which were sympatric and with little geographic structure. Nuclear markers (ITS2 and Wingless) showed little variation and gave no indications that *E. autumnata* comprises more than one species. The samples were screened with primers for *Wolbachia* outer surface gene (*wsp*) and 12% of the samples tested positive. Two *Wolbachia* strains were associated with different mtDNA sub-clades within *E. autumnata,* which may indicate indirect selection/selective sweeps on haplotypes. Our results demonstrate that deep mtDNA divergences are not synonymous with cryptic speciation and this has important implications for the use of mtDNA in species delimitation, like in DNA barcoding.

## Introduction

Species are often regarded as basic units of evolution and correct species delimitation serves as a backbone in most biological studies (Mayr 1982; Roe and Sperling 2007). However, the number of described species is a small portion of the estimated extant number and there is a need for an increased ability to identify and discriminate species (Blaxter 2004; Silva-Brandao et al. 2009). For the last three decades, mitochondrial DNA has been extensively used (Ballard and Whitlock 2004) and proven to be an important tool in species delimitation as it possesses biological properties making it suitable as a marker for molecular biodiversity (Moore 1995; Hebert et al. 2003).

A universal system for rapid, inexpensive species identification applicable for any life stage, DNA barcoding, has been proposed by Hebert et al. (2003). The ambition behind DNA barcoding is identification by sequencing of short standardized gene regions in order to assign unknown individuals to species and to enhance the discovery of new species. The assumptions underlying DNA barcoding are that every species have sets of unique barcode sequences and hence constitutes monophyletic clades and that genetic variation between species exceeds the variation within species (Hebert et al. 2003). Nevertheless, there are examples of deep intraspecific divergences in mtDNA, also in sympatric populations of animal groups such as birds (Omland et al. 2000; Johnsen et al. 2010; Hogner et al. in press), beetles (Schulenburg et al. 2002; Avtzis et al. 2008), and spiders (Chang et al. 2007). There are several possible explanations for high intraspecific variation. First, this pattern may reflect the presence of cryptic species. The exploration of cryptic species within the Skipper butterfly, *Astraptes fulgerator*, performed by Hebert et al. (2004) is a well-known example. By combining DNA barcoding with information about ecology and morphology of *A. fulgerator*, at least 10, largely sympatric cryptic species were revealed (but see Brower 2006). Second, demographic effects like isolation will cause differentiation between isolated populations by the accumulation of mutations over time. The differentiation may then reflect early stages of speciation. Secondary admixture of allopatrically evolved populations will in many cases result in gene trees with pronounced phylogenetic gaps between branches (Avise 2000). However, haplotype loss due to genetic drift (i.e., lineage sorting) will over time make a population monophyletic for a single gene lineage (Beebee and Rowe 2004). As lineage sorting is more prominent in small populations, the number of haplotypes maintained in a population is a function of current and historic effective population sizes. In closely related species, allele fixation often fails to complete and they will in these cases share ancestral polymorphisms resulting in discordance between gene trees and species trees (Moore 1995; Beebee and Rowe 2004). For nuclear DNA, when reproductive barriers do not evolve in allopatry and if secondary contact is obtained, variation must be maintained by factors opposing gene flow (e.g., geography and ecology). This is because gene flow will homogenize the nuclear genome over time (Futuyma 2005). Third, introgression by hybridization between closely related species can cause mtDNA to show a different gene genealogy than most other genes in the species in question. As the gene genealogy resulting from introgression is very similar to that expected by ancestral polymorphism and incomplete linage sorting (Ballard and Whitlock 2004), distinguishing between isolation and ancient hybridization can be very difficult. Finally, interpretation of mitochondrial genetic diversity may be hampered by the presence of heritable endoparasitic symbionts and in some cases result in incongruence between nDNA and mtDNA (Linares et al. 2009). Among the most widespread are bacteria from the genus *Wolbachia* (Alphaproteobacteria: Rickettsiales) (Russell et al. 2009). It has long been recognized that endoparasitic symbionts are prevalent among arthropods and that these organisms may have an important role in arthropod evolution as they can cause a number of reproductive alterations in their host, the most common being cytoplasmic incompatibility (Rousset et al. 1992; Werren 1997; Hurst et al. 1999; Hurst and Jiggins 2005; Narita et al. 2009). Male-killing parthenogenesis and feminization of genetic males are other alterations documented in arthropods (Rousset et al. 1992; Grandjean et al. 1993; Werren et al. 1995; Werren 1997; Jiggins 2003; Hurst and Jiggins 2005). The effects of inherited symbionts can be mistaken as evidence for population structure and admixture, as an mtDNA genealogy with deep internal branches could be the result of multiple selective sweeps from different *Wolbachia* strains, rather than a population being large and old or because of secondary admixture (Hurst and Jiggins 2005). Nevertheless, analysis and comparison of sequence data from both mtDNA and nDNA should help distinguishing between demographic effects and indirect selection on mtDNA by parasitic bacteria in an infected population (Rokas et al. 2001; Raychoudhury et al. 2010).

**Figure 1:**
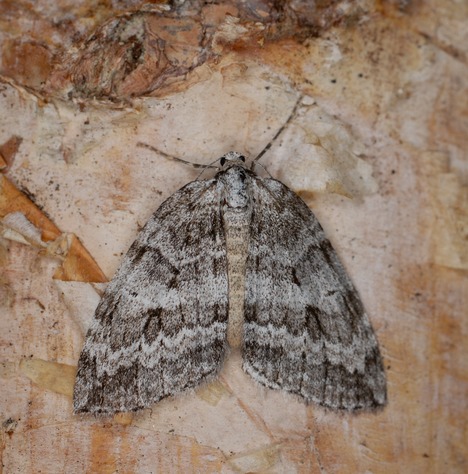
The study species, *Epirrita autumnata*. Photo: Svein Bekkum.

The genus *Epirrita* constitutes nine species (Scoble 1999), of which three are distributed in Norway (Aarvik et al. 2009). These are the autumnal moth, *Epirrita autumnata,* pale November moth, *Epirrita christyi,* and November moth, *Epirrita dilutata*. *E. autumnata* (Fig. 1) is distributed from Japan and Manchuria through Mongolia, Siberia, and Caucasus, to Western Europe and from the northern parts of Scandinavia to the Mediterranean (Skou 1984). The subspecies *E*. *autumnata omissa* and *E*. *autumnata henshawi* are found in North America (Tenow 1972; Scoble 1999). The larvae feed on deciduous trees, especially on birch (*Betula*), alder (*Alnus*), and willow (*Salix)* and have cyclic outbursts with ∼9- to10-year intervals (Tenow 1972; Aarvik et al. 2009). In periods with high larvae densities, it can defoliate and seriously harm the mountain birch (*Betula pubescens* ssp*. czerepanovii*) forests (Ruohomaki et al. 2000; Jepsen et al. 2008; Yang et al. 2008). As a consequence of the moths' cyclical population dynamics, northern populations of *E. autumnata* may experience present-day bottlenecks as outbreaks are followed by collapse in population size and subsequent decline in genetic variability. Hence, one might expect to find relatively low levels of genetic variation within this species (Futuyma 1998; Snäll et al. 2004). However, preliminary results from DNA barcoding of Scandinavian moths and butterflies (Lepidoptera) revealed discrepancy between present division to species and sequence divergence in the genus *Epirrita* (Johnsen, Aarvik & Lifjeld, unpublished data). In particular, high sequence variation clustered in several well-defined haplogroups within sympatric *E. autumnata* suggested that this might be a complex of cryptic species.

The main aims of this study were to examine the relatively high mtDNA variation found within *E. autumnata*, describe the degree of sympatry among haplogroups within Norwegian populations of this species and compare the variation at mitochondrial (Cytochrome *c* Oxidase subunit 1, CO1) and nuclear (Internal Transcribed Spacer 2, ITS2 and Wingless) loci. In particular, we wanted to investigate four possible explanations for high intraspecific mtDNA variation: (1) presence of cryptic species; (2) historic isolation and secondary contact; (3) introgression from a related species; and (4) *Wolbachia* infections associated with different haplogroups. First, if the high mtDNA diversity reflects cryptic species, we predict congruence between divergence in mtDNA and nDNA sequence data, given that there has been sufficient time for divergence. Second, if the pattern is due to isolation and secondary contact, we predict higher differentiation in mtDNA compared with nDNA because the former has a relatively high evolutionary rate (5–10 times higher than single copy nDNA) (Avise 1986). Furthermore, depending on the amount of time since range expansions and secondary contact, we expect some degree of mtDNA- and nDNA structure, reflecting the demographic history and original geographic distribution of the lineages, again with higher degree of structure in mtDNA. Third, if ancient introgression by hybridization caused the differentiation in *E. autumnata* mtDNA, the same predictions as for historic isolation with secondary contact will apply. However, if introgression occurred recently, we would expect to find overlapping haplotypes with closely related species (e.g., *E. dilutata* and/or *E. christyi*). Finally, if *Wolbachia* infections have affected the mtDNA variation within this species, we predict an association between infection status and haplogroups and incongruence between mtDNA and nDNA. The samples were screened for *Wolbachia* infections to evaluate whether *Wolbachia* might have influenced patterns of mitochondrial diversity in *E. autumnata*.

## Material and methods

### Material examined

A total of 87 moths from the genus *Epirrita* were examined in the course of this study, of which 79 were collected in Norway, five in Finland, and three in Scotland (Appendix, Table 4). The Norwegian moths were sampled from different parts of Norway in the period 1999–2009. The middle leg of each moth was collected and stored in ethanol for DNA extraction and the abdomen was removed from some of the specimens for the purpose of making genital preparations. The rest of the animal was prepared dry and pinned as voucher. Information about the samples is available at the Barcode of Life Data Systems website (http://www.boldsystems.org) in the “NorBOL – Lepidoptera – Epirrita” project. In addition, two Wingless and three *wsp* sequences (see below) were downloaded from GenBank and included in the analysis. Sequences downloaded from GenBank are identified by accession numbers in the phylogenetic trees.

### Genetic analysis

#### DNA extraction

Legs were dried at 50°C and transferred to eppendorf tubes. To speed up lysis, the legs were crushed into smaller pieces. DNA extraction was carried out using the E.Z.N.A tissue kit (Omega Bio-tek Inc, Norcross, GA), according to the manufacturer's protocol. The lysis reaction proceeded overnight and the DNA elution was performed with 100-*μ*L elution buffer.

#### Amplification

Amplification of a 658 base pair long COI fragment from the COI 5′ region was performed using the primers Lep-F1 (5′-ATTCAACCAATCATAAAGATAT-3′; Hebert et al. 2004) and Lep-R1, (5′-TAAACTTCTGGATGTCCAAAAA-3′ Hebert et al. 2004). In cases where these primers failed to amplify, a second reverse primer EnhLep-R1 (5′-CTCCWCCAGCAGGATCAAAA-3′; Hajibabaei et al. 2006) was used in combination with Lep-F1, targeting a 612-bp fragment of the COI region. The PCR profile used for this marker was as follows: 94°C for 1 min, 94°C for 30 sec, 46°C for 40 sec, 72°C for 1 min, (step 2–4 cycled 5 times), 94°C for 30 sec, 51°C for 40 sec, 72°C for 1 min, (step 5–7 cycled 35 times), and 72°C for 10 min.

A 500- to 514-bp long fragment, depending on the species, from the ITS2 region was amplified using the forward primer ITS3b (5′-GGGTCGATGAAGAACGCAST-3′; Roe and Sperling 2007) and reverse primer ITS4 (5′-TCCTCCGCTTATTGATATGC-3′; White et al. 1990). If these primers failed to amplify, another forward primer, FFA (5′-TGTGAACTGCAGGACACA-3′, Brown et al. 2000) was used. PCR profiles for the ITS2 markers were as follows: 94°C for 2 min, 94°C for 30 sec, 55°C for 1 min, 72°C for 2 min, (step 2-4 cycled 34 times), and 72°C for 10 min.

To amplify a 408-bp long fragment from the Wingless region, a single primer pair was used; LepWG1_f (5′-GARTGYAARTGYCAYGGYATGTCTGG-3′; Brower and DeSalle 1998) and LepWG2_r (5′-ACTICGCRCACCARTGGAATGTRCA-3′; Brower and DeSalle 1998). The PCR profile was as follows: 95°C for 5 min, 95°C for 1 min, 50°C for 1 min, 72°C for 2 min, (step 2–4 cycled 35 times) and 72°C for 10 min. For some of the Wingless samples, more than one fragment was amplified. In these cases, a second electrophoresis was performed using 7-*μ*L PCR product and 4-*μ*L loading dye. The PCR product was cut out with scalpel under UV light, cleaned up, and DNA was extracted following the protocol NucleoSpin^**®**^ Extract II, PCR clean-up/extraction kit (Macherey-Nagel, Düren, Germany). To solubilize the gel slices, 200 *μ*L NT buffer pr. 100 *μ*g gel/PCR product was used.

General *wsp* primers were used to amplify 555–560 bp, depending on the strain, from the *Wolbachia* outer surface gene; *wsp* 81F (5′-TGG TCC AAT AAG TGA TGA AGAAAC-3′; Braig et al. 1998) and *wsp* 691R (5′-AAA AAT TAA ACG CTA CTC CA-3′; Braig et al. 1998). The following PCR profile was used with the *wsp* primers: 94°C for 1 min, 94°C for 30 sec, 55°C for 40 sec, 72°C for 1 min, (step 2–4 cycled 35 times) and 72°C for 10 min. All *wsp* sequences were cloned in case of multiple infections (see below).

PCR reactions were performed in 10- or 12.5-*μ*L reaction volume. The final concentration of the various chemicals was as follows: 1× buffer, 1.5mM MgCl_2_, 0.8mM dNTPs, 0.5 mM of the forward and reverse primers, 3% DMSO, 1U/*μ*L Platinum Taq DNA Polymerase (Invitrogen, Carlsbad, CA), and dH_2_O to make up the remaining reaction volume. The DNA template had a final concentration of 15–50 ng. The MgCl_2_ concentration and/or polymerase concentration was increased when no bands were visible with agarose gel screening. All samples were screened on 1% agarose gel, stained with ethidiumbromide or SYBR- Safe (Invitrogen). *Wolbachia* screenings were performed with positive control. In cases where no bands were visible, a second amplification/screening was performed to confirm the result.

#### Cloning of *wsp* sequences

Cloning was performed following the TOPO10 Cloning protocol (Invitrogen). The PCR product was heated to 68°C for 10 min before the TOPO cloning reaction was set up. We used 2-*μ*L PCR product and let the reaction incubate for 15 min at room temperature. We used *E.coli* DH5α cells for transformation and the transformed cells were transferred to growth medium (LB agar) containing Kanamycin (100 *μ*g/mL) as selection marker. DNA from 6–8 clones from each individual were picked out and diluted in 6-*μ*L dH_2_O (for two individuals only three colonies were obtained, however, they all gave the same result). The samples were then amplified and sequenced as described below using standard M13 primers.

#### Sequencing

The samples were cleaned for unconsumed primers and nucleotides using Exo-Sap-IT (United States Biochemical, Cleveland, OH), diluted 10 times and incubated at 37°C for 45 min for degradation of excess primers and nucleotides and inactivated at 80°C for 15 min. Cycle sequencing was performed in 10-μl reaction volume, using BigDye v3.1 cycle sequencing kit with 5× BigDye Terminator sequencing buffer (Applied Biosystems, Foster City, CA) and a program following manufacturer's recommendations. Purification was performed using ethanol/EDTA/sodium acetate precipitation. Electrophoresis and data analysis of samples were performed with an ABI 3130×l capillary electrophoresis instrument.

The four regions were sequenced in both directions and the resulting consensus sequences were aligned by ClustalW and manually edited in MEGA version 4.0.2 (Tamura et al. 2007). Using highly conserved primers, there is a risk of co-amplifying non-functional copies of mtDNA (numts) in addition to the targeted mtDNA and numts have been shown to be a source of error by overestimation of unique species inferred from the analysis (Gellissen et al. 1983; Lopez et al. 1994; Song et al. 2008). Careful examination of the sequences can reveal numts based on properties such as indels, frameshift mutations, in-frame stop codons, unexpected nucleotide composition, and systematic double peaks (Song et al. 2008). Alignments generated from the three coding regions (COI, Wingless, and *wsp*) were translated from nucleotide- to amino acid sequences to check for stop codons and frameshift mutations.

### Phylogenetic and statistical analyses

A model test was performed on all four data sets using MEGA5 version 5.05 (Tamura et al. 2011*)* to find the best fit substitution models for the different markers. Neighbor-joining analyses, calculation of genetic distances, and standard errors between the different haplogroups were performed in MEGA5 using the Tamura 3-parameter (Tamura 1992) (COI and *wsp*) – and the Jukes–Cantor algorithm (Jukes and Cantor 1969) (ITS2 and Wingless) with all sites included, the complete deletion option, assuming homogenous pattern among lineages and uniform substitution rates among sites. Bootstrap values were calculated in MEGA5 using 10 000 iterations.

To test for neutrality, DnaSP version 5.10 (Librado and Rozas 2009) was used to compute Tajima's *D* (Tajima 1989). This test is based on the allele frequency spectrum and can be used to infer previous evolutionary and demographic events in the population. Positive values indicate an excess of intermediate-frequency alleles, which might result from balancing selection or bottlenecks, while negative values reflect an excess of rare polymorphisms, which might result from positive selection or a population expansion (Akey et al. 2004). We also calculated the two common measures of nucleotide polymorphism, π, the average number of nucleotide differences per site between two sequences and θ, the population mutation parameter estimated from the number of segregating sites in the aligned sample of sequences (Nei 1987).

Analysis of molecular variance (AMOVA: Excoffier et al. 1992) and calculation of F_ST_ (Wright 1951) were performed on 53 *E. autumnata* COI sequences of the Norwegian samples using ARLEQUIN version 3.5.1.2 (Excoffier et al. 2005), to investigate how genetic variation was distributed within and between regions. The 14 Norwegian sampling locations were divided into four regions: north (*N* = 5), east (*N* = 22), south (*N* = 13), and west (*N* = 13) (Appendix, Table 4). The analysis was conducted with pairwise difference as distance method.

## Results

### Mitochondrial and nuclear DNA variation

Translation from nucleotide- to amino acid sequences of the analyzed regions revealed no stop codons, frameshifts, or systematic double peaks and the mtDNA base composition was as expected, with a high A-T content (68%) (Perna and Kocher 1995).

**Figure 2:**
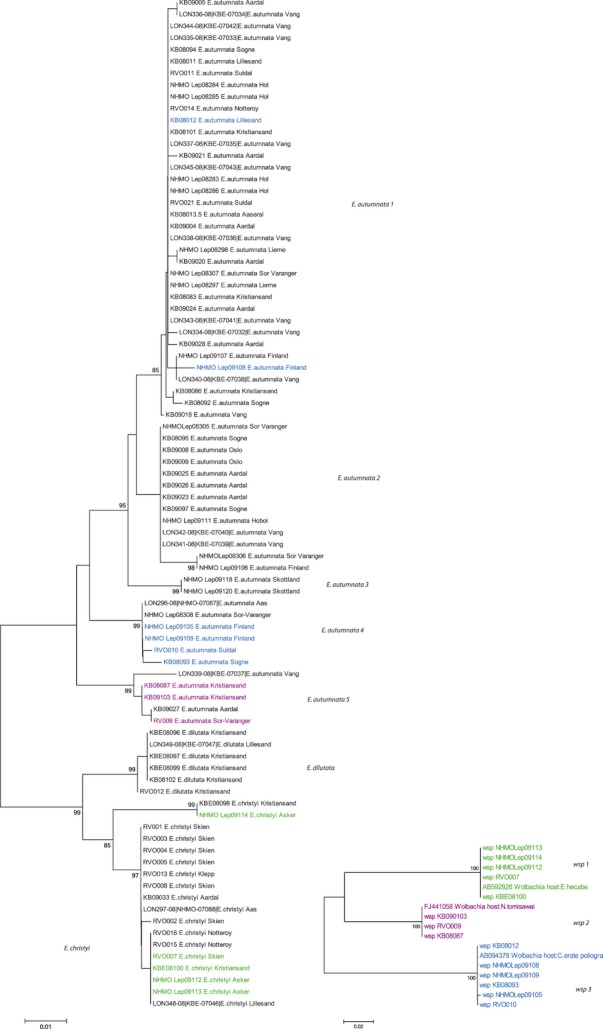
(a) Neighbor-joining analysis of 86 *Epirrita* samples based on the COI gene (Tamura 3-parameter used as substitution model)*. E. dilutata* and *E. christyi* are included as out-groups. The five monophyletic groups of distinct *E. autumnata* haplotypes show high intraspecific variation. Bootstrap support (10000 iterations) is shown at each node. (b) Neighbor-joining analysis based on the *wsp* gene (Tamura 3-parameter used as substitution model). Bootstrap support (10000 iterations) is indicated at each node. Infections caused by bacteria represented in *wsp* group 1 are found in *E. christyi*. Infections from *wsp* group 2 are found in *E. autumnata* haplogroup 5, while infections represented in *wsp* group 3 are found in *E.* *autumnata* group 1 and 4. For each clade, we have added a representative *wsp* sequence (downloaded from Genbank) that has been identified in other Lepidoptera species (*Eurema hecabe**,** Nephopterix tomisawai, Colias erate* subsp. *polygraphus*).

**Figure 3:**
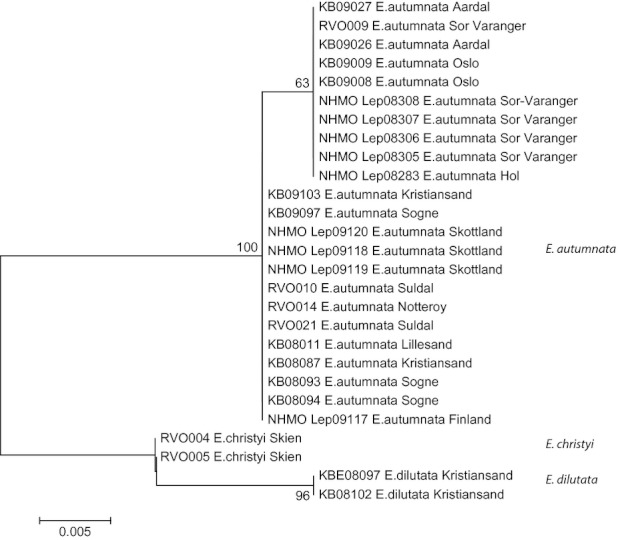
Neighbor-joining analysis of 27 *Epirrita* sequences based on the ITS2 gene (Jukes–Cantor used as substitution model). *E. christyi* and *E. dilutata* are included as out-groups. *E.* *autumnata* are shown as one monophyletic group with some diversity (< 0.5%). Bootstrap support (10000 iterations) is shown at each node.

**Figure 4:**
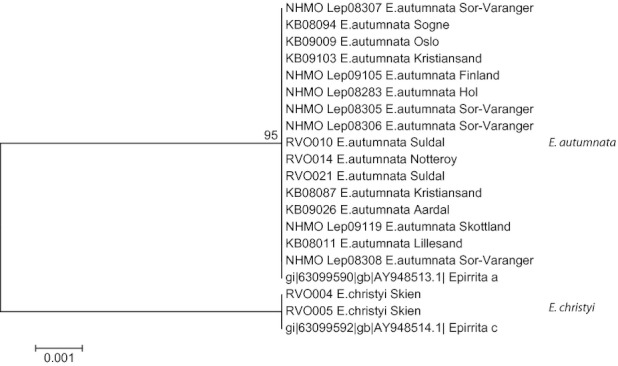
Neighbor-joining analysis of 20 *Epirrita* sequences based on the Wingless gene and with *E. christyi* as out-group (Jukes–Cantor used as substitution model). Bootstrap support (10,000 iterations) is shown at each node.

**Figure A1:**
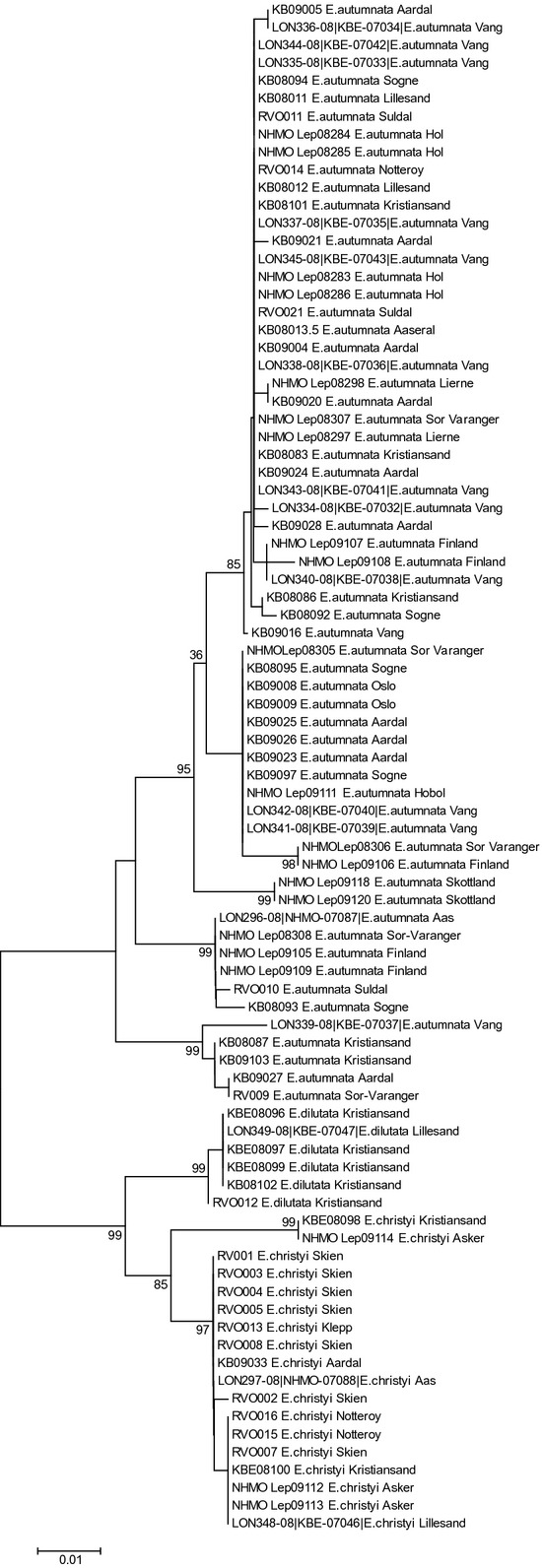
Minimum evolution analysis (Tamura 3-parameter as substitution model) of the COI data set (n = 86), showing the same structure as the NJ analysis, with 5 distinct COI-haplotypes. Bootstrap (10000 iterations) is shown at each node.

**Figure A2:**
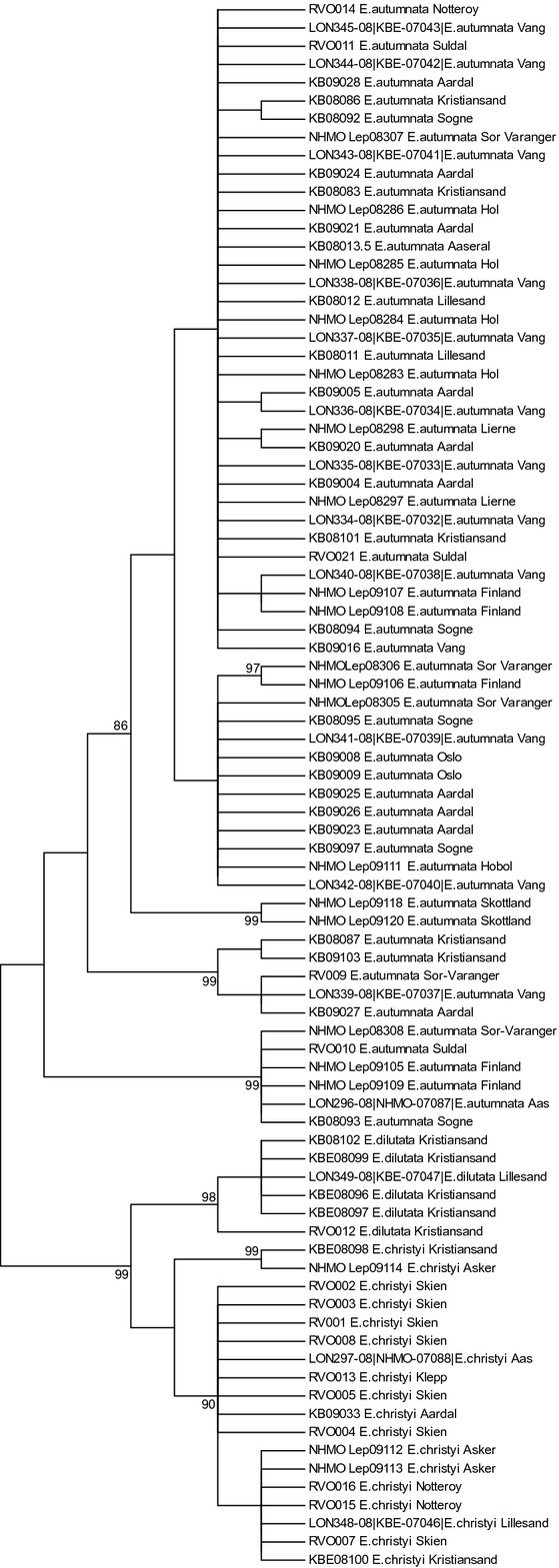
Maximum parsimony analysis (using the complete deletion option) of the COI data set (n = 86). MP analysis also shows high intraspecific variation within E. autumnata. Bootstrap values (500 iterations) are shown at each node.

Neighbor-joining analysis of the COI data set showed high intraspecific variation within *E. autumnata*, with 21 haplotypes divided into five distinct haplogroups with varying degree of support at each node, ranging from 63% to 99% (Fig. 2a, only bootstrap values higher than 85% are shown). Standard estimates of nucleotide polymorphism were higher within *E. autumnata* than within *E. christiy* and *E. dilutata* (Table 1). Genetic distance between *E. autumnata* COI haplogroups 1–5 ranged from 1.5% to 4.1% (Table 2). Assuming a COI substitution rate of 1.5–2.3% per million years (Brower 1994; Farrell 2001; Kandul et al. 2004), genetic distance as high as 4.1% (distance between haplogroup 1 and 5) suggests divergence as far back as 1.7–2.7 million years. Interspecific distances among *E. autumnata*, *E. christyi,* and *E. dilutata* ranged from 2.9 to 7.6%. Haplogroup 3 consists of moths from Scotland, while the remaining four groups comprise samples from all four Norwegian regions: north, east, south, and west. This shows a high degree of sympatry of mtDNA linages in the northern *E. autumnata* populations. The Neighbor-joining topology based on the COI data is supported by Minimum evolution and Maximum parsimony analysis generated in MEGA5 (Appendix Fig. A1 and A2). In contrast, the phylogenies based on nuclear loci show far less intraspecific variation. In the phylogenies based on the ITS2- and Wingless data sets, *E. autumnata* constitutes one monophyletic group with ∼0.5% and no variation, respectively (Fig. 3 and 4). The AMOVA reveals that COI haplotype variation is much higher within regions (98.3%), than between regions (1.7%) (Table 3; overall F_ST_ = 0.017, *P* = 0.27). Estimates of Tajima's *D* were negative, but not significantly different from zero for COI in all three species (Table 1).

**Table 1:** Polymorphism statistics for *Epirrita autumnata* (and *Epirrita christyi* and *Epirrita dilutata* for COI) from the COI, Wingless and ITS2 region, and from *Wolbachia* outer surface gene (*wsp*)

	Species	N^1^	L^2^	Tajima's D^3^	π^4^	θ^5^
COI	*E. autumnata*	62	658	-0.77659	0.01674	0.02043
	*E. christyi*	18	658	-0.89359	0.00693	0.00907
	*E. dilutata*	6	658	-0.93302	0.00058	0.00076
Wingless	*E. autumnata*	14	409	-1.15524	0.00039	0.00085
ITS2	*E. autumnata*	23	484	1.99999	0.00285	0.542
*wsp*	*E. autumnata*	17	564	-0.93302	0.10812	0.06573

1 Number of individuals.

2 Sequence length.

3 None of the *D* values significant.

4 Average pairwise sequence difference per nucleotide (Nei 1987).

5 Expected heterozygosity per nucleotide (Watterson 1975).

**Table 2:** Genetic distance (Tamura 3-parameter) between the five *Epirrita autumnata* haplotype groups 1-5 (below diagonal) with standard errors (above diagonal)

	E. a 1	E. a 2	E. a 3	E. a 4	E. a 5
*E. autumnata1*		0.005	0.007	0.009	0.010
*E. autumnata2*	0.015		0.007	0.008	0.009
*E. autumnata3*	0.023	0.022		0.009	0.009
*E. autumnata4*	0.035	0.033	0.039		0.009
*E. autumnata5*	0.041	0.036	0.040	0.037	

**Table 3:** AMOVA of Norwegian *Epirrita autumnata* samples showing haplotype distribution in four selected regions in Norway; north, east, south, and west

Source of variation	d.f	Sum of squares	Variance components	Percentage of variation
Among regions	3	14.444	0.069 Va	1.710
Within regions	49	194.273	3.965 Vb	98.290
Total	52	208.717	4.034	

### *Wolbachia* screening

Of the 71 samples screened, 17 (12%) tested positive for *Wolbachia*. It was possible to generate *wsp* sequences from 14 of the 17 infected samples and all 14 individuals had only one *wsp* sequence as revealed by cloning. Hence, there was no case of multiple infections. Comparing these sequences with sequences in GenBank matched strains found in various insect taxa, including Lepidoptera (99% match). NJ analysis of the 14 sequences obtained using *wsp* primers combined with sequences downloaded from GenBank, splits the sequences into three clusters with 100% bootstrap support at each node (Fig. 2b). Infections from bacteria in *wsp* group 1 were only found in *E. christyi*, whereas *wsp* group 2 and 3 were found exclusively in *E. autumnata*. Interestingly, the two *wsp* groups infecting *E. autumnata* were associated with different haplogroups: *wsp* group 2 occurred only in *E. autumnata* haplogroup 5, whereas *wsp* group 3 occurred in haplogroups 1 and 4. Given the observed frequency ratio of 1:2 for the two *wsp* groups in *E. autumnata*, the probability that the *Wolbachia* positives within each of three haplogroups (*N* = 2, 3 and 4, respectively) would not show mixed *wsp* genotypes can be estimated to *P* = 0.039. Hence, we conclude that there was a significant association between *Wolbachia* strains and *E. autumnata* haplogroups. Screening for *Wolbachia* also revealed fragments in *E. autumnata* haplogroup 2 (2 individuals). The origin of these bands is unknown as no sequences were obtained from the PCR products. However, it would be interesting to sequence these bands in a future study as they might consist of a more divergent *Wolbachia* strain that requires other suitable primers.

## Discussion

We found five distinct mtDNA haplogroups within *E. autumnata* in northern Europe, but little variation was found in the nuclear regions ITS2 and Wingless. High degree of sympatry and little geographic structure in *E. autumnata* haplotype distribution was evident. Twelve percent of the screened samples proved to be infected with *Wolbachia* and there was a close association between particular COI haplogroups within *E. autumnata* and the different *Wolbachia* strains.

### Mitochondrial and nuclear incongruence

Combining data sets from unlinked genes should be informative in questions regarding species delineation, as unlinked genes are expected to have independent genealogical histories (Maddison 1997). In this study, genetic analysis of gene regions from different genomes (mtDNA and nDNA) gives different estimates of intraspecific variation within *E. autumnata*. The COI region reveals high sympatric intraspecific divergence (Fig. 2a) with genetic distances ranging from 1.5% to 4.1% (Table 2). One might argue that an intraspecific genetic distance of 4% is not high compared with distances found within other taxa. For example, there are several studies on land snails that show a higher degree of intraspecific divergence than that found within *E. autumnata*. However, many of these examples concern isolated and/or morphologically distinct populations (Hayashi and Chiba 2000; Shimizu and Ueshima 2000; Bond et al. 2001; Pinceel et al. 2005). In this context, we want to emphasize that the divergent *E. autumnata* haplogroups occur sympatrically and that the level of genetic distance within *E. autumnata* is comparable to the level of divergence commonly seen between sister species in Lepidoptera (Huemer and Hausmann 2009; Lukhtanov et al. 2009; Hajibabaei 2006; this study). In contrast to the levels of intraspecific variation found in COI, the ITS2 and Wingless regions show little (∼0.5%) and no variation, respectively (Fig. 3 and 4).

The presence of cryptic species has been suggested to explain high intraspecific divergence in several studies (Hebert et al. 2004; Roe and Sperling 2007; Vaglia et al. 2008). However, the presence of cryptic species predicts divergence in both mtDNA and nDNA and the results from this study show clear incongruence between the two data sets. In addition, preliminary analyses show no obvious intraspecific variation in genital structures and no association between flight period and haplogroups (Kvie & Aarvik, unpublished data). These findings all imply that cryptic speciation is not a likely explanation for high intraspecific mtDNA variation within *E. autumnata*. Nevertheless, using nuclear markers that evolve faster and that are more variable than Wingless and ITS2 might generate a different result than we found in this study. It is a well-known challenge to find nuclear markers that evolve fast enough to separate between cryptic species (Dasmahapatra and Mallet 2006). However, there are examples of studies performed on closely related- and cryptic arthropod species that have used these nuclear markers successfully (Roe and Sperling 2007; Schmitz et al. 2007; Linares et al. 2009; Dincă et al. 2011; Sun et al. 2011).

As the COI data set implies divergence as far back as 1.7–2.7 million years, a possible hypothesis would be separation of *E. autumnata* into different glacial refugia in Pleistocene (2 – 0.01 million years ago). It is a common perception that many extant sister taxa diverged during the cyclic climate in this period (Avise and Walker 1998; Avise 2000; Beebee and Rowe 2004). If the variation found in northern *E. autumnata* mtDNA is a result of separation into several refugia, we would expect some degree of geographic separation restricting gene flow. However, results from the AMOVA (Table 3) shows that most of the genetic variation is found within (98.3%) and not between populations (1.7%). Also, if mtDNA variation in the northern populations is a result of isolation, and we are looking at early stages of speciation, variation should be detectable in both mitochondrial- and nuclear markers (Jiggins and Tinsley 2005). As analysis of *E. autumnata* nDNA only reveals small amounts of variation and the results from the AMOVA show a small degree of variation between the populations, it is not likely that isolation alone can explain the high mtDNA variation found in this study.

There are European studies showing opposing results. Snäll et al. (2004) analyzed the mtDNA control region investigating the dispersal of *E. autumnata* females and differentiation between northern- (Norwegian samples) and southern (Finnish samples) *E. autumnata* populations. They found less variation in northern- compared with southern populations, which they argue might be a result of the northern moths' cyclic population dynamics (Futuyma 1998; Snäll et al. 2004). Their results also revealed moderate levels of divergence between the northern and southern populations. In addition, results from a study performed by Hausmann et al. (2011) showed no variation within Bavarian *E. autumnata* and the published sequences from that project cluster with moths in haplogroup 2 from this study (data not shown). As the two Scottish samples from our study also cluster together in one group (haplogroup 3, Fig. 2a), it is likely that there is a geographic structure at a larger scale and more samples from a wider range should be investigated.

There are no indications of hybridization between the Norwegian *Epirrita* species as no shared haplotypes between *E. christyi*, *E. dilutata,* and *E. autumnata* were found. However, ancient introgression by hybridization or introgression from another extant congeneric cannot be ruled out because this will give similar gene genealogy as ancestral polymorphisms caused by isolation (Ballard and Whitlock 2004). It should be noted that in Lepidoptera, females are the heterogametic sex and that according to Haldane's rule (Haldane 1922), hybrid sterility/inviability will be more severe in the heterogametic sex, thereby reducing the likelihood of heterospecific mtDNA introgression (but see Zakharov et al. 2009). Besides, even if both isolation and ancient introgression by hybridization could explain the origin of intraspecific variation in *E. autumnata*, neither of these processes can explain the high degree of incomplete linage sorting still existing in *E. autumnata* mtDNA. Geography, ecology, and reproductive barriers are all factors that could maintain variation within a species, but we did not find evidence for any of these factors playing a role in this study. Using nuclear markers that evolve faster, performing more thorough morphometric examinations of genitalia, and testing for other ecological and morphological differences like host plant preference and larvae differentiation could give a more solid basis for concluding about these possibilities.

### Association between *Wolbachia* infections and COI haplogroups

Screening for *Wolbachia* infections showed infections in *E. autumnata* and in *E. christyi* (Fig. 2a and 2b). This result, combined with the results from NJ analysis of the COI region (Fig. 2a) and the AMOVA (Table 3), resembles those of Schulenburg et al. (2002). They examined Eurasian two-spot ladybirds, *Adalia bipunctata,* infected with endoparasites from the genera *Rickettsia* and *Spiroplasma,* in addition to infections by two distinct strains of *Wolbachia*. Also in this case, did mtDNA sequence analysis show an association between infection status and distribution of haplotypes, but no association between haplotype and geography. However, Shoemaker et al. (2004) showed that *Wolbachia*-infected species tend to have lower levels of mtDNA diversity than uninfected closely related species. Reduced levels of variation are the most commonly documented effect in *Wolbachia*-infected populations (Shoemaker et al. 1999; Dean et al. 2003; Jiggins 2003; Shoemaker et al. 2004). Nevertheless, high levels of diversity in mtDNA may be maintained within a population when infected with bacteria of different strains, as different strains might cause selective sweeps on different haplotypes. The diversity will, in these cases, depend on the number of symbionts the population harbors (Hurst and Jiggins 2005). Symbionts like *Wolbachia* are also known to cause hybrid introgression and possibly balancing selection on cytoplasmic genes and may therefore be an important factor in creating variation within a population or in a species (Jiggins 2003). For example, Jiggins and Tinsley (2005) found significantly elevated levels of mtDNA diversity in infected *Adalia bipunctata* beetles. They argued that the effects of endoparasitic symbionts can be considerably more complex than simple reduction in diversity following a selective sweep. As several samples in this study tested positive for *Wolbachia* and there seems to be an association between haplogroups in *E. autumnata* and infection class, it is possible that the mitochondrial genome of *E. autumnata* has undergone several *Wolbachia* infections and subsequent selective sweeps, maintaining the diversity within this species. However, as the test of selection based on Tajima's *D* gave a non-significant result, we cannot rule out the possibility that drift rather than selective sweeps causes variation to be maintained in this species. Some mtDNA haplotypes and their associated *Wolbachia* variants might be carried to high frequencies because of the cyclical fluctuations in population size in *E. autumnata*.

### DNA barcoding Lepidoptera

DNA barcoding has proven to be a useful tool for species identification in a wide range of animal species, including Lepidoptera (Hebert et al. 2004; Hajibabaei 2006; Silva-Brandao et al. 2009; Hausmann et al. 2011, but see Elias et al. 2007; Wiemers and Fiedler 2007). This study shows that sequencing the barcode region is sufficient for discriminating between specimens of Norwegian moths in the genus *Epirrita*, hence fulfilling one of the main objectives of DNA barcoding (species identification of unknown specimens; Hebert et al. 2003). However, our results also demonstrate that delimiting species based on mtDNA divergence alone, whether based on a threshold distance, monophyly, or diagnostic nucleotides (Moritz and Cicero 2004; van Velzen et al. 2012), may lead to erroneous conclusions and inflation of species numbers, supporting previous critiques of the species discovery aspect of DNA barcoding (Moritz and Cicero 2004; Hickerson et al. 2006). It is becoming increasingly clear that integrating information from several independent genetic loci as well as morphological and/or ecological variation is required for defining new species (DeSalle et al. 2005; Galtier et al. 2009; Damm et al. 2010; Dupuis et al. 2012; Towes and Brelsford 2012). As such, DNA barcoding can be a useful method for initial screening of biodiversity, to discover interesting genetic variation worthy of further study.

### Concluding remarks

Analysis of the COI region reveals high divergence within *E. autumnata* compared with the nuclear regions. As 12% of the samples surveyed in this study tested positive for *Wolbachia,* the COI data set should be interpreted with care. Our analyses revealed no association between the distribution of mitochondrial haplotypes and geography. Nevertheless, ecological and morphological factors should be examined more thoroughly to rule out the possibility of the different haplogroups reflecting early stages of speciation. As there seems to be an association between *Wolbachia* infections and mtDNA haplogroups, a likely explanation for the divergences in *E.* *autumnata* mtDNA is that current populations consist of separate lineages that once evolved in allopatry, without evolving reproductive barriers. At some point, secondary contact is obtained and gene flow reduces variation in the nuclear genome over time, while *Wolbachia* infections contribute to maintain the variation in the mitochondrial genome. The effect of linage sorting also seems prominent as there is one dominant haplogroup (haplogroup 1, Fig. 2a).

From these findings, we conclude that current taxonomy is correct and it is probable that *Wolbachia* contributes to intraspecific mtDNA variation by maintaining less common lineages that normally would have been sorted out**.**

## Data accessibility

DNA sequences, GenBank accession: COI: JX260731-JX260816, ITS2: JN225584-JN225610, Wingless: JN225566-JN225583, Wsp: JX310335-JX310348.

## Appendix

**Table A1:** Information about the samples surveyed in this study, sampling locations, coordinates and collecting dates. Footnotes behind the municipalities show how the Norwegian *E. autumnata* samples were grouped into four Norwegian regions (used in the AMOVA)

Tissue sample	Species	Country	Municipality	Coordinates	Collecting date
NHMO Lep08305	*Epirrita autumnata*	Norway	Sør-Varanger^1^	69°33′48.53″N, 30°05′11.19″E	10-20.VIII.2008
NHMO Lep08306	*Epirrita autumnata*	Norway	Sør-Varanger^1^	69°27′09.34″N, 30°03′28.97″E	9.IX-2.X.2008
NHMO Lep08307	*Epirrita autumnata*	Norway	Sør-Varanger^1^	69°33′48.53″N, 30°05′11.19″E	10-20.VIII.2008
NHMO Lep08308	*Epirrita autumnata*	Norway	Sør-Varanger^1^	69°33′48.53″N, 30°05′11.19″E	10-20.VIII.2008
RVO009	*Epirrita autumnata*	Norway	Sør-Varanger^1^	69°22′14.45″N,29°40′43.56″E	3.VIII-25.IX.2006
NHMO Lep08297	*Epirrita autumnata*	Norway	Lierne^2^	64°26′44.32″N, 13°42′57.43″E	25.IX.2008
NHMO Lep08298	*Epirrita autumnata*	Norway	Lierne^2^	64°26′44.32″N, 13°42′57.43″E	25.IX.2008
NHMO Lep08283	*Epirrita autumnata*	Norway	Hol^2^	60°31′33.79″N, 8°18′21.14″E	5-7.IX.2008
NHMO Lep08284	*Epirrita autumnata*	Norway	Hol^2^	60°31′33.79″N, 8°18′21.14″E	5-7.IX.2008
NHMO Lep08285	*Epirrita autumnata*	Norway	Hol^2^	60°31′33.79″N, 8°18′21.14″E	5-7.IX.2008
NHMO Lep08286	*Epirrita autumnata*	Norway	Hol^2^	60°31′33.79″N, 8°18′21.14″E	5-7.IX.2008
RVO014	*Epirrita autumnata*	Norway	Nøtterøy^2^	59°12′20.38″N, 10°33′49.53″E	5.VIII.2006
KB09008	*Epirrita autumnata*	Norway	Oslo^2^	59°53′52.18″N, 10°43′55.37″E	4-23.IX.2008
KB09009	*Epirrita autumnata*	Norway	Oslo^2^	59°53′52.18″N, 10°43′55.37″E	21.VIII-4.IX.2008
NHMO Lep09111	*Epirrita autumnata*	Norway	Hobøl^2^	59°38′30.02″N, 10°59′50.52″E	25-26.IX.2009
KB09016	*Epirrita autumnata*	Norway	Vang^2^	61°09′29.51″N, 8°31′40.88″E	IX.2007
KBE07032	*Epirrita autumnata*	Norway	Vang^2^	61°09′29.51″N, 8°31′40.88″E	1.IX.2006
KBE07033	*Epirrita autumnata*	Norway	Vang^2^	61°09′29.51″N, 8°31′40.88″E	1.IX.2006
KBE07034	*Epirrita autumnata*	Norway	Vang^2^	61°09′29.51″N, 8°31′40.88″E	1.IX.2006
KBE07035	*Epirrita autumnata*	Norway	Vang^2^	61°09′29.51″N, 8°31′40.88″E	1.IX.2006
KBE07036	*Epirrita autumnata*	Norway	Vang^2^	61°09′29.51″N, 8°31′40.88″E	1.IX.2006
KBE07037	*Epirrita autumnata*	Norway	Vang^2^	61°09′29.51″N, 8°31′40.88″E	1.IX.2006
KBE07038	*Epirrita autumnata*	Norway	Vang^2^	61°09′29.51″N, 8°31′40.88″E	1.IX.2006
KBE07039	*Epirrita autumnata*	Norway	Vang^2^	61°09′29.51″N, 8°31′40.88″E	1.IX.2006
KBE07040	*Epirrita autumnata*	Norway	Vang^2^	61°09′29.51″N, 8°31′40.88″E	1.IX.2006
KBE07041	*Epirrita autumnata*	Norway	Vang^2^	61°09′29.51″N, 8°31′40.88″E	1.IX.2006
KBE07042	*Epirrita autumnata*	Norway	Vang^2^	61°09′29.51″N, 8°31′40.88″E	1.IX.2006
KBE07043	*Epirrita autumnata*	Norway	Vang^2^	61°09′29.51″N, 8°31′40.88″E	1.IX.2006
NHMO Lep07087	*Epirrita autumnata*	Norway	Ås^2^	60°04′02.28″N, 10°11′15.06″E	27.IX.2007
KB08011	*Epirrita autumnata*	Norway	Lillesand^3^	58°11′07.17″N,8°13′58.90″E	4.X.2007
KB08012	*Epirrita autumnata*	Norway	Lillesand^3^	58°11′07.17″N,8°13′58.90″E	X.2007
KB08013,5	*Epirrita autumnata*	Norway	Åseral^3^	58°47′11.51″N,7°19′12.63″E	11.X.2007
KB08092	*Epirrita autumnata*	Norway	Søgne^3^	58°07′37.47″N,7°36′40.09″E	7.X.2008
KB08093	*Epirrita autumnata*	Norway	Søgne^3^	58°07′37.47″N,7°36′40.09″E	17.X.2008
KB08094	*Epirrita autumnata*	Norway	Søgne^3^	58°07′37.47″N,7°36′40.09″E	17.X.2008
KB08095	*Epirrita autumnata*	Norway	Søgne^3^	58°07′37.47″N,7°36′40.09″E	17.X.2008
KB09097	*Epirrita autumnata*	Norway	Søgne^3^	58°07′37.47″N,7°36′40.09″E	17.X.2008
KB08083	*Epirrita autumnata*	Norway	Kristiansand^3^	58°09′39.95″N, 8°05′57.44″E	29.IX.2008
KB08086	*Epirrita autumnata*	Norway	Kristiansand^3^	58°09′39.95″N, 8°05′57.44″E	6.X.2008
KB08087	*Epirrita autumnata*	Norway	Kristiansand^3^	58°12′08.76″N, 8°06′05.92″E	12.X.2008
KB08101	*Epirrita autumnata*	Norway	Kristiansand^3^	58°12′08.76″N, 8°06′05.92″E	18.X.2008
KB09103	*Epirrita autumnata*	Norway	Kristiansand^3^	58°12′08.76″N, 8°06′05.92″E	12.X.2008
KB09004	*Epirrita autumnata*	Norway	Årdal^4^	61°21′19.81″N,7°52′47.72″E	5-15.IX.2008
KB09005	*Epirrita autumnata*	Norway	Årdal^4^	61°21′19.81″N,7°52′47.72″E	5-15.IX.2008
KB09020	*Epirrita autumnata*	Norway	Årdal^4^	61°21′19.81″N,7°52′47.72″E	IX.2008
KB09021	*Epirrita autumnata*	Norway	Årdal^4^	61°21′19.81″N,7°52′47.72″E	IX.2008
KB09023	*Epirrita autumnata*	Norway	Årdal^4^	61°21′19.81″N,7°52′47.72″E	IX.2008
KB09024	*Epirrita autumnata*	Norway	Årdal^4^	61°21′19.81″N,7°52′47.72″E	X.2008
KB09025	*Epirrita autumnata*	Norway	Årdal^4^	61°21′19.81″N,7°52′47.72″E	X.2008
KB09026	*Epirrita autumnata*	Norway	Årdal^4^	61°21′19.81″N,7°52′47.72″E	X.2008
KB09027	*Epirrita autumnata*	Norway	Årdal^4^	61°21′19.81″N,7°52′47.72″E	X.2008
KB09028	*Epirrita autumnata*	Norway	Årdal^4^	61°21′19.81″N,7°52′47.72″E	X.2008
RVO021	*Epirrita autumnata*	Norway	Suldal^4^	59°29′49.34″N, 6°15′30.65E”	2.X.2002
RVO010	*Epirrita autumnata*	Norway	Suldal^4^	59°32′50.25″N,6°23′13.76″E	18.X.2004
RVO011	*Epirrita autumnata*	Norway	Suldal^4^	59°39′27.09″N,6°52′06.80″E	24.X.2002
RVO013	*Epirrita christyi*	Norway	Klepp	58°44′23.49″N, 5°30′45.48″E	14.X.2001
RVO015	*Epirrita christyi*	Norway	Nøttery	59°12′20.38″N, 10°33′49.53″E	IX.2006
RVO016	*Epirrita christyi*	Norway	Nøttery	59°12′20.38″N, 10°33′49.53″E	IX.2006
RVO001	*Epirrita christyi*	Norway	Skien	59°08′00.51″N, 9°39′25.22″E	11.IX.2008
RVO002	*Epirrita christyi*	Norway	Skien	59°08′00.51″N, 9°39′25.22″E	11.IX.2008
RVO003	*Epirrita christyi*	Norway	Skien	59°08′00.51″N, 9°39′25.22″E	11.IX.2008
RVO004	*Epirrita christyi*	Norway	Skien	59°08′00.51″N, 9°39′25.22″E	11.IX.2008
RVO005	*Epirrita christyi*	Norway	Skien	59°08′00.51″N, 9°39′25.22″E	11.IX.2008
RVO007	*Epirrita christyi*	Norway	Skien	59°08′00.51″N, 9°39′25.22″E	11.IX.2008
RVO008	*Epirrita christyi*	Norway	Skien	59°08′00.51″N, 9°39′25.22″E	11.IX.2008
KB09033	*Epirrita christyi*	Norway	Årdal	61°21′19.81″N, 7°52′47.72″E	17.X.2008
KBE08098	*Epirrita christyi*	Norway	Kristiansand	58°09′39.95″N, 8°05′57.44″E	18.X.2008
KBE08100	*Epirrita christyi*	Norway	Kristiansand	58°12′08.76″N, 8°06′05.92″E	18.XI.2008
NHMO Lep09112	*Epirrita christyi*	Norway	Asker	59°50′10.26″N, 10°28′01.80″E	1.X.2009
NHMO Lep09113	*Epirrita christyi*	Norway	Asker	59°50′10.26″N, 10°28′01.80″E	1.X.2009
NHMO Lep09114	*Epirrita christyi*	Norway	Asker	59°50′10.26″N, 10°28′01.80″E	1.X.2009
NHMO Lep07088	*Epirrita christyi*	Norway	Ås	60°04′02.28″N, 10°11′15.06″E	27.IX.2007
KBE07046	*Epirrita christyi*	Norway	Lillesand	58°11′07.17″N,8°13′58.90″E	30.IX.2007
KB08102	*Epirrita dilutata*	Norway	Kristiansand	58°12′08.76″N, 8°06′05.92″E	28.X.2008
KBE08096	*Epirrita dilutata*	Norway	Kristiansand	58°12′08.76″N, 8°06′05.92″E	18.X.2008
KBE08097	*Epirrita dilutata*	Norway	Kristiansand	58°12′08.76″N, 8°06′05.92″E	18.X.2008
KBE08099	*Epirrita dilutata*	Norway	Kristiansand	58°12′08.76″N, 8°06′05.92″E	18.X.2008
RVO012	*Epirrita dilutata*	Norway	Kristiansand	58°04′06.92″N, 7°58′52.55″E	1.XI.1999
KBE07047	*Epirrita dilutata*	Norway	Lillesand	58°11′07.17″N,8°13′58.90″E	14.X.2007
NHMO Lep09105	*Epirrita autumnata*	Finland	Lohja	60°15′01.16″N, 24°04′45.68″E	29.IX.2008
NHMO Lep09106	*Epirrita autumnata*	Finland	Lohja	60°15′01.16″N, 24°04′45.68″E	29.IX.2008
NHMO Lep09107	*Epirrita autumnata*	Finland	Lohja	60°15′01.16″N, 24°04′45.68″E	29.IX.2008
NHMO Lep09108	*Epirrita autumnata*	Finland	Hyvinkââ	60°37′54.63″N, 24°51′51.13″E	8.X.2008
NHMO Lep09109	*Epirrita autumnata*	Finland	Lohja	60°15′01.16″N, 24°04′45.68″E	29.IX.2008
NHMO Lep09118	*Epirrita autumnata*	Scotland	Banffshire	57°25′11.28″N, 2°38′35.08″V	18.X.2009
NHMO Lep09119	*Epirrita autumnata*	Scotland	Banffshire	57°25′11.28″N, 2°38′35.08″V	18.X.2009
NHMO Lep09120	*Epirrita autumnata*	Scotland	Banffshire	57°25′11.28″N, 2°38′35.08″V	18.X.2009

1 North.

2 East.

3 South.

4 West.

## Appendix

**Table A2:** GenBank accession numbers

Tissue sample	Species	COI	ITS2	Wingless	*wsp*
NHMO Lep08305	*Epirrita autumnata*	JX260769	JN225585	JN225572	
NHMO Lep08306	*Epirrita autumnata*	JX260785	JN225586	JN225573	
NHMO Lep08307	*Epirrita autumnata*	JX260741	JN225587	JN225566	
NHMO Lep08308	*Epirrita autumnata*	JX260738	JN225588	JN225581	
RVO009	*Epirrita autumnata*	JX260749	JN225606	JN225568	JX310341
NHMO Lep08297	*Epirrita autumnata*	JX260733			
NHMO Lep08298	*Epirrita autumnata*	JX260786			
NHMO Lep08283	*Epirrita autumnata*	JX260759	JN225584	JN225571	
NHMO Lep08284	*Epirrita autumnata*	JX260775			
NHMO Lep08285	*Epirrita autumnata*	JX260783			
NHMO Lep08286	*Epirrita autumnata*	JX260782			
RVO014	*Epirrita autumnata*	JX260772	JN225590	JN225575	
KB09008	*Epirrita autumnata*	JX260758	JN22600		
KB09009	*Epirrita autumnata*	JX260768	JN225601		
NHMO Lep09111	*Epirrita autumnata*	JX260754			
KB09016	*Epirrita autumnata*	JX260757			
KBE07032	*Epirrita autumnata*	JX260784			
KBE07033	*Epirrita autumnata*	JX260739			
KBE07034	*Epirrita autumnata*	JX260760			
KBE07035	*Epirrita autumnata*	JX260762			
KBE07036	*Epirrita autumnata*	JX260752			
KBE07037	*Epirrita autumnata*	JX260771			
KBE07038	*Epirrita autumnata*	JX260773			
KBE07039	*Epirrita autumnata*	JX260789			
KBE07040	*Epirrita autumnata*	JX260788			
KBE07041	*Epirrita autumnata*	JX260756			
KBE07042	*Epirrita autumnata*	JX260743			
KBE07043	*Epirrita autumnata*	JX260753			
NHMO Lep07087	*Epirrita autumnata*	JX260779			
KB08011	*Epirrita autumnata*	JX269737	JN225592	JN225580	
KB08012	*Epirrita autumnata*	JX260787			JX310345
KB08013,5	*Epirrita autumnata*	JX260778			
KB08092	*Epirrita autumnata*	JX260745			
KB08093	*Epirrita autumnata*	JX260736	JN225595		JX310346
KB08094	*Epirrita autumnata*	JX260744	JN225596	JN225567	
KB08095	*Epirrita autumnata*	JX260742			
KB09097	*Epirrita autumnata*	JX260780	JN225604		
KB08083	*Epirrita autumnata*	JX260770			
KB08086	*Epirrita autumnata*	JX260751			
KB08087	*Epirrita autumnata*	JX260791	JN225593	JN225577	JX310342
KB08101	*Epirrita autumnata*	JX260746			
KB09103	*Epirrita autumnata*	JX260740	JN225605	JN225569	JX310340
KB09004	*Epirrita autumnata*	JX260731			
KB09005	*Epirrita autumnata*	JX260761			
KB09020	*Epirrita autumnata*	JX260790			
KB09021	*Epirrita autumnata*	JX260765			
KB09023	*Epirrita autumnata*	JX260767			
KB09024	*Epirrita autumnata*	JX260766			
KB09025	*Epirrita autumnata*	JX260792			
KB09026	*Epirrita autumnata*	JX260774	JN225602	JN225578	
KB09027	*Epirrita autumnata*	JX260734	JN225603		
KB09028	*Epirrita autumnata*	JX260732			
RVO021	*Epirrita autumnata*	JX260747	JN225591	JN225576	
RVO010	*Epirrita autumnata*	JX260710	JN225589	JN225574	JX310348
RVO011	*Epirrita autumnata*	JX260781			
RVO013	*Epirrita christyi*	JX260808			
RVO015	*Epirrita christyi*	JX260796			
RVO016	*Epirrita christyi*	JX260802			
RVO001	*Epirrita christyi*	JX260800			
RVO002	*Epirrita christyi*	JX260806			
RVO003	*Epirrita christyi*	JX260794			
RVO004	*Epirrita christyi*	JX260807	JN225607	JN225582	
RVO005	*Epirrita christyi*	JX260793	JN225608	JN225583	
RVO007	*Epirrita christyi*	JX260799			JX310336
RVO008	*Epirrita christyi*	JX260797			
KB09033	*Epirrita christyi*	JX260804			
KBE08098	*Epirrita christyi*	JX260809			
KBE08100	*Epirrita christyi*	JX260805			JX310335
NHMO Lep09112	*Epirrita christyi*	JX260803			JX310337
NHMO Lep09113	*Epirrita christyi*	JX260810			JX310338
NHMO Lep09114	*Epirrita christyi*	JX260795			JX310339
NHMO Lep07088	*Epirrita christyi*	JX260798			
KBE07046	*Epirrita christyi*	JX260801			
KB08102	*Epirrita dilutata*	JX260814	JN225610		
KBE08096	*Epirrita dilutata*	JX260813			
KBE08097	*Epirrita dilutata*	JX260816	JN225609		
KBE08099	*Epirrita dilutata*	JX260812			
RVO012	*Epirrita dilutata*	JX260811			
KBE07047	*Epirrita dilutata*	JX260815			
NHMO Lep09105	*Epirrita autumnata*	JX260764	JN225594	JN225570	JX310347
NHMO Lep09106	*Epirrita autumnata*	JX260735			
NHMO Lep09107	*Epirrita autumnata*	JX260763			
NHMO Lep09108	*Epirrita autumnata*	JX260748			JX310343
NHMO Lep09109	*Epirrita autumnata*	JX260777			JX310344
NHMO Lep09118	*Epirrita autumnata*	JX260750	JN225598		
NHMO Lep09119	*Epirrita autumnata*		JN225599	JN225579	
NHMO Lep09120	*Epirrita autumnata*	JX260776	JN225597		
